# Single‐cell profiling reveals distinct immune phenotypes that contribute to ischaemia‐reperfusion injury after steatotic liver transplantation

**DOI:** 10.1111/cpr.13116

**Published:** 2021-09-01

**Authors:** Xinyu Yang, Di Lu, Rui Wang, Zhengxing Lian, Zuyuan Lin, Jianyong Zhuo, Hao Chen, Modan Yang, Winyen Tan, Mengfan Yang, Xuyong Wei, Qiang Wei, Shusen Zheng, Xiao Xu

**Affiliations:** ^1^ Department of Hepatobiliary and Pancreatic Surgery The Center for Integrated Oncology and Precision Medicine Affiliated Hangzhou First People's Hospital Zhejiang University School of Medicine Hangzhou China; ^2^ Zhejiang University Cancer Center Hangzhou China; ^3^ Department of Hepatobiliary and Pancreatic Surgery The First Affiliated Hospital Zhejiang University School of Medicine Hangzhou China; ^4^ NHC Key Laboratory of Combined Multi‐organ Transplantation Hangzhou China; ^5^ Institute of Organ Transplantation Zhejiang University Hangzhou China; ^6^ Department of Hepatobiliary and Pancreatic Surgery Shulan (Hangzhou) Hospital Hangzhou China

**Keywords:** fatty graft, ischaemia‐reperfusion injury, liver transplantation, single‐cell RNA‐sequencing

## Abstract

**Objectives:**

The discrepancy between supply and demand of organ has led to an increased utilization of steatotic liver for liver transplantation (LT). Hepatic steatosis, however, is a major risk factor for graft failure due to increased susceptibility to ischaemia‐reperfusion (I/R) injury during transplantation.

**Materials and methods:**

To assess the plasticity and phenotype of immune cells within the microenvironment of steatotic liver graft at single‐cell level, single‐cell RNA‐sequencing (scRNA‐Seq) was carried out on 23 675 cells from transplanted rat livers. Bioinformatic analyses and multiplex immunohistochemistry were performed to assess the functional properties, transcriptional regulation, phenotypic switching and cell‐cell interactions of different cell subtypes.

**Results:**

We have identified 11 different cell types in transplanted livers and found that the highly complex ecosystem was shaped by myeloid‐derived cell subsets that transit between different states and interact mutually. Notably, a pro‐inflammatory phenotype of Kupffer cells (KCs) with high expression of colony‐stimulating factor 3 (CSF3) that was enriched in transplanted steatotic livers was potentially participated in fatty graft injury. We have also detected a subset of dendritic cells (DCs) with highly expressing XCR1 that was correlated with CD8^+^ T cells, mediating the severer steatotic liver damage by I/R injury.

**Conclusions:**

The findings of our study provide new insight into the mechanisms by which steatosis exacerbates liver damage from I/R injury. Interventions based on these observations create opportunities in attenuating fatty liver graft injury and expanding the donor pool.

## Graphical abstract

The schematic of workflow for the sample preparation, sequencing and bioinformatic analysis.

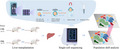

## 1 INTRODUCTION

Liver transplantation is a life‐saving intervention for patients with end‐stage liver diseases. The increasing gap between the demand and supply of organs has extended the interest in utilizing expanded criteria donors (ECD) to expand the liver donor pool.

With the rising prevalence of obesity, metabolic syndrome and type 2 diabetes, incidence of non‐alcoholic fatty liver disease (NAFLD) has also increased concomitantly, affecting around 25% of the population worldwide.^1^, ^2^, ^3^ The growing prevalence of NAFLD results in a multifaceted impact on transplant recipients, in which steatotic livers at the early stage of NAFLD accounted for about 25% of donors for liver transplantation.^4^ An analysis from the United Network for Organ Sharing (UNOS) database has also shown that NAFLD reduces the liver donor pool with the estimated fall of overall liver graft utilization from 78% to 44% in 2030.^5^

Steatotic liver is a common type of ECD. However, such organs are more prone to preservation injury and graft dysfunction, resulting in a poor prognosis after liver transplantation.^6^, ^7^, ^8^ It has been proposed that steatotic livers are more susceptible to ischaemia‐reperfusion (I/R) injury, which impairs liver regeneration and is a major cause of liver damage.^6^, ^9^ The transcriptional regulatory network during disease progression remains to be explored as the underlying cellular and molecular mechanisms of fatty liver transplant failure are still poorly understood.

Recent development of single‐cell RNA‐sequencing (scRNA‐Seq) technologies has allowed comprehensively profiling the organ ecosystem and understanding the cellular heterogeneity of disease in an unprecedented way. Application of scRNA‐Seq on immune cell populations has identified novel immune subsets in many diseases.^10^, ^11^, ^12^ Hence, we conducted a comprehensive scRNA‐Seq on cells from transplanted livers without filtering the cell type markers. Interrogation into the organ ecosystem revealed a considerable heterogeneity and plasticity in diverse cell types especially in immune‐related cells. Importantly, we have also detected and validated a new subset of CSF3^+^ Kupffer cells (KCs) that was correlated with progression of transplant‐associated injury in liver transplantation using fatty liver graft. Moreover, we found an increased fractions of dendritic cells (DCs) and CD8^+^ T cells in the immune component of fatty liver donor (FDL) group. Interestingly, DCs in FDL, which was characterized by overexpression of XCR1, presented an efficient antigen‐presenting ability primarily and played a crucial role in linking the innate and adaptive immune response. These results promote the understanding of heterogeneity between different sources of donor and provide a basis for individualized treatment for fatty graft in liver transplantation.

## 2 MATERIALS AND METHODS

### 2.1 Animals

Under specific pathogen‐free conditions, Sprague Dawley (SD) rats were purchased from the Zhejiang Academy of Medical Sciences. Four weeks of SD rats were used for donors. We built two types of grafts through different diets: the donors in fatty graft group (n = 3/group) were fed with high‐fat diet (D12492) for 8 weeks to produce moderate fatty liver,^13^, ^14^ whereas the donors in normal graft group (n = 3/group) and all the recipient rats were fed with normal diet. All animals were given free access to water. The rats were housed in a standard animal laboratory with free activity and access to water and chow. They were kept under specific pathogen‐free (SPF) conditions with a 12‐hour light and dark cycle. All operations were performed under clean conditions. All animal studies were conducted in accordance with the National Institute Guide for the Care and Use of Laboratory Animals. The experimental protocols were authorized by the Ethics Committee for the Use of Experimental Animals in Zhejiang University.

### 2.2 Experimental design

Rat orthotopic liver transplantation was conducted in two groups: (i) control donor liver (CDL group: n = 3) and (ii) fatty donor liver (FDL group: n = 3). Each group consisted of three liver transplants. Liver tissue and blood of six recipients were sampled at the time of 24 hours after liver transplantation.

### 2.3 Rat orthotopic liver transplantation procedure

In the model of orthotopic liver transplantation (OLT), SD rats with body weight of about 400 g were used. The surgery was carried out according to Kamada's 2‐cuff methods.^15^ Briefly, the donor rat was subjected to anaesthesia and subsequent systemic heparinization. The donor liver detached from the rat was immersed in University of Wisconsin (UW) solution and then was implanted orthotopically into the abdomen of the recipient rat. The anastomosis of the suprahepatic vena cava was continuously sutured with 8‐0 microscopic vascular suture. The cuff technique was applied to connect portal vein and infrahepatic vena cava. After then, the bile duct was reconstructed by an end‐to‐end anastomosis over an indwelling stent. Hepatic artery was not reconstructed. After OLT, standard rodent chow and sterilized water were available ad libitum.

### 2.4 Single‐cell dissociation

Single‐cell RNA‐seq experiment was performed by experimental personnel in the laboratory of GENECHEM. The tissues were surgically removed and kept in MACS Tissue Storage Solution (Miltenyi Biotec) until processing. The tissue samples were processed as described below. Briefly, samples were first washed with phosphate‐buffered saline (PBS), minced into small pieces (approximately 1 mm^3^) on ice and enzymatically digested with 100 U/mL collagenase IV (Worthington) and 30 U/mL DNase I (Worthington) for 45 minutes at 37°C, with agitation. After digestion, samples were sieved through a 70 μm cell strainer and centrifuged at 300 g for 5 minutes. After the supernatant was removed, the pelleted cells were suspended in red blood cell lysis buffer (Miltenyi Biotec) to lyse red blood cells. After washing with PBS containing 0.04% BSA, the cell pellets were re‐suspended in PBS containing 0.04% BSA and re‐filtered through a 40 μm cell strainer. Dissociated single cells were then stained for viability assessment using Calcein‐AM (Thermo Fisher Scientific) and Draq7 (BD Biosciences). The single‐cell suspension was further enriched with a MACS dead cell removal kit (Miltenyi Biotec).

### 2.5 Single‐cell RNA‐sequencing

BD Rhapsody system was used to capture the transcriptomic information of the liver tissue single cells. Single‐cell capture was achieved by random distribution of a single‐cell suspension across >200 000 microwells through a limited dilution approach. Beads with oligonucleotide barcodes were added to saturation so that a bead was paired with a cell in a microwell. The cells were lysed in the microwell to hybridize mRNA molecules to barcoded capture oligos on the beads. Beads were collected into a single tube for reverse transcription and ExoI digestion. Upon cDNA synthesis, each cDNA molecule was tagged on the 5′ end (that is, the 3′ end of a mRNA transcript) with a unique molecular identifier (UMI) and cell barcode indicating its cell of origin. Whole transcriptome libraries were prepared using the BD Rhapsody single‐cell whole‐transcriptome amplification (WTA) workflow including random priming and extension (RPE), RPE amplification PCR and WTA index PCR. The libraries were quantified using a High Sensitivity DNA chip (Agilent) on a Bioanalyzer 2200 and the Qubit High Sensitivity DNA assay (Thermo Fisher Scientific). Sequencing was performed by illumina sequencer (Illumina) on a 150 bp paired‐end run.

### 2.6 Quality control and unsupervised clustering of cells

We applied fastp with default parameter filtering the adaptor sequence and removed the low‐quality reads (Table S2).^16^ Unique Molecular Identifier (UMI) tools were applied for single‐cell transcriptome analysis to identify the cell barcode whitelist, extract the cell barcode UMIs and calculate the cell expression counts based on the filtered clean fastq data.^17^ With the data of scRNA‐Seq, unsupervised clustering of cells was performed with Seurat package version 3.2.3. Genes were filtered out if these expressed in less than two cells. Cells with >200 genes and <20% of mitochondrial genes were further processed. Then, Seurat arithmetic was used to calculate the variation coefficient of genes. Based on the first 10 000 highest alterable genes, principal part analysis (PCA) was used to perform dimensionality reduction of all data. A k‐nearest neighbour graph was executed with Euclidean distances in the space of the first 10 significant principal components. The Louvain Modularity optimization algorithm clustered the cells in the picture, and then the results of unsupervised clustering were visualized by using t‐distributed stochastic neighbour embedding (t‐SNE) and uniform manifold approximation and projection (UMAP) projects.

### 2.7 Identification of marker genes and cell‐type annotation

Cells were classified into the major cell types using SingleR (v1.4).^18^ Ten major cell types (Endothelial cells, Hepatocytes, Macrophages, Kupffer cells (KCs), conventional and plasmacytoid dendritic cells (cDCs and pDCs, respectively), NK cells, Monocytes, T cells, B cells and Granulocytes) remained after excluding those cells expressing fewer than 200 genes. Each subgroup object underwent the same dimensionality reduction, clustering and visualization approach as described above. Each subgroup object was then further split into clusters and manually annotated with known cell type markers (Table S1).

### 2.8 Cell trajectory analysis

To map differentiation of myeloid‐derived cells, pseudotime analysis was performed with Monocle2 to determine the dramatic translational relationships among cell types and clusters.^19^ Only top 3000 variable genes identified by differentialGeneTest were used for analysis. Then, branch expression analysis modelling (BEAM) was used to identify genes with branch‐dependent expression. Further detection with the Monocle2 plot_genes_branched_heatmap function revealed the key role of a series of genes in the differentiation progress.

### 2.9 Pathway analysis

Differentially expressed genes (DEGs) of cell subgroups were recognized by the findmarker function provided by Seurat. |FC| > 1 and adj.*P*.val <.05 were used as the cut‐off criteria. Functional enrichment analysis was performed on these DEGs with clusterProfiler. The genes from significantly enriched pathways were used as new gene sets for further Gene Set Variation Analysis (GSVA). Differences between different cell groups were calculated with a linear model offered by FindMarkers in Seurat package.

### 2.10 Cell‐cell communication analysis with CellPhoneDB 2

CellPhoneDB v.2.0 is a Python‐based computational analysis tool to analyse interactions between pairwise cell clusters,^20^ which includes a public repository of curated ligands, receptors and their interactions. Firstly, we identified the FDL‐related significant genes in each targeted cell type as candidate genes for ligand‐receptor (L‐R). Then, we ran the CellPhoneDB framework using a statistical method and detected L‐R pairs that were expressed in more than 5% of cells.

### 2.11 Single‐cell RNA‐seq data portal

Interactive analysed data with searchable functions have been visualized in an online resource—the scRNA‐Seq Atlas of Fatty Liver Graft Injury after Liver Transplantation: scRNA‐FLGILT (https://ciopm.shinyapps.io/scrna‐flgilt/). scRNA‐FLGILT has features including: visualization of cell type atlas and summative and comparative gene analysis expression. The data portal template was developed using R Shiny framework.

### 2.12 Histological analysis

Tissue specimens from rat liver grafts were fixed in 4% formalin, embedded in paraffin and stored in 4°C. For further examination, the specimens were sliced into 5‐μm‐thick sections and stained with haematoxylin‐eosin (H&E) and oil‐red‐O (ORO) staining. Two independent investigators examined all tissue section in a blinded fashion. The non‐alcoholic fatty liver disease (NAFLD) activity score was calculated using the existing scoring system.^21^ The severity of IRI was graded using Suzuki's criteria, which depends on the degree of congestion, vacuolization and necrosis.^22^ The scores were evaluated in 10 random fields (magnification ×200) per slide and averaged for each slide.

### 2.13 Terminal deoxynucleotidyl transferase‐mediated dUTP nick end labelling assay

DNA fragments in liver sections, resulting from necrosis/apoptosis, were detected by an in situ apoptosis detection kit (11684817; Roche). Terminal deoxynucleotidyl transferase‐mediated dUTP nick end labelling (TUNEL) analysis was performed according to the kit's instructions.

### 2.14 Serum transaminase assay and lipid detection

Rat serum ALT, AST, γ‐GT, TC, TG and LDL‐C levels were detected using Fully Automatic Biochemical Analyzer (BS‐220; Mindray).

### 2.15 Multiplexed immunofluorescence staining

Multiplex staining of formalin‐fixed paraffin‐embedded (FFPE) tissue was performed after deparaffinize and rehydrate, antigen retrieval, spontaneous fluorescence quenching and BSA blocking. CLEC4F (Cat# AF2784; Novus biologicals) and CSF3 (Cat# DF9542; Affinity Biosciences) antibodies were sequentially applied, followed by species‐corresponded secondary antibody incubation. Nuclei were stained with DAPI after all the antigens had been labelled. The stained slides were scanned to obtain multispectral images using the confocal microscope (Olympus). For each slide, 5 fields of immune cell‐enriched tumoural area were selected for image capture.

### 2.16 Statistical analysis

Cell distribution comparisons between two groups were performed using unpaired two‐tailed Wilcoxon rank‐sum tests. Comparisons of gene expression or gene signature between two groups of cells were performed using unpaired two‐tailed Student's *t* test. All statistical analyses and graph generation were performed in r (version 3.6.0) and graphpad prism (version 8.0).

## 3 RESULTS

### 3.1 scRNA‐Seq profiling of the microenvironment in transplanted livers

**FIGURE 1:**
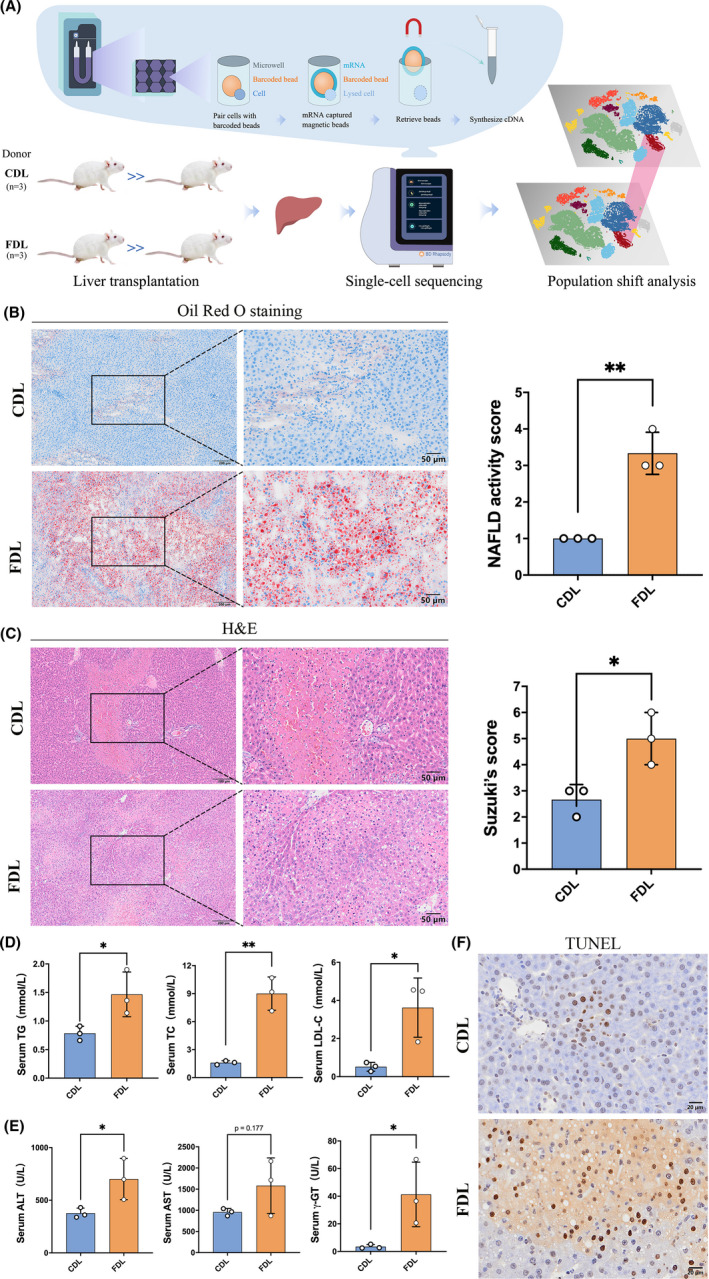
Liver steatosis affects graft function in rat orthotopic liver transplantation (OLT). (A) Schematic of workflow for the sample preparation, sequencing and bioinformatic analysis. (B) Histological staining of hepatic tissue by oil‐red‐O and the quantification of NAFLD activity score. (C) Histological staining of hepatic tissue by H&E and the quantification of the Suzuki's score. (D) Serum lipid levels (TC, TG and LDL‐C) were measured at 24 h after reperfusion. (n = 3 per group). (E) Serum ALT, AST and γ‐GT levels were measured at 24 h after reperfusion. (n = 3 per group). (F) Representative TUNEL staining of liver sections from CDL or FDL group at 24 h after reperfusion. ALT, alanine aminotransferase; AST, aspartate aminotransferase; CDL, control donor liver; FDL, fatty donor liver; LDL‐C, low‐density lipoprotein cholesterol; TC, total cholesterol; TG, triglyceride; TUNEL, terminal transferase‐mediated dUTP nick end labelling; γ‐GT, γ‐glutamyl transferase. *P < .05; **P < .01. All data are shown as the mean ± SD

To determine the distinctive cellular populations and mediators across different forms of donor grafts, we generated single‐cell suspensions from transplanted liver tissues after LT using CDL (n = 3) and FDL (n = 3), and performed scRNA‐Seq using the BD Rhapsody platform (see Materials and Methods and Figure 1A). Meanwhile, H&E and Oil‐red O staining combined with serum lipid detections provide robust evidence for the success of fatty liver development (Figure 1B,D). We next assessed the degree of damage in transplanted livers between CDL and FDL. H&E, TUNEL staining and liver function detection altogether showed severer hepatocellular damage in FDL (Figure 1C,E,F).

**FIGURE 2:**
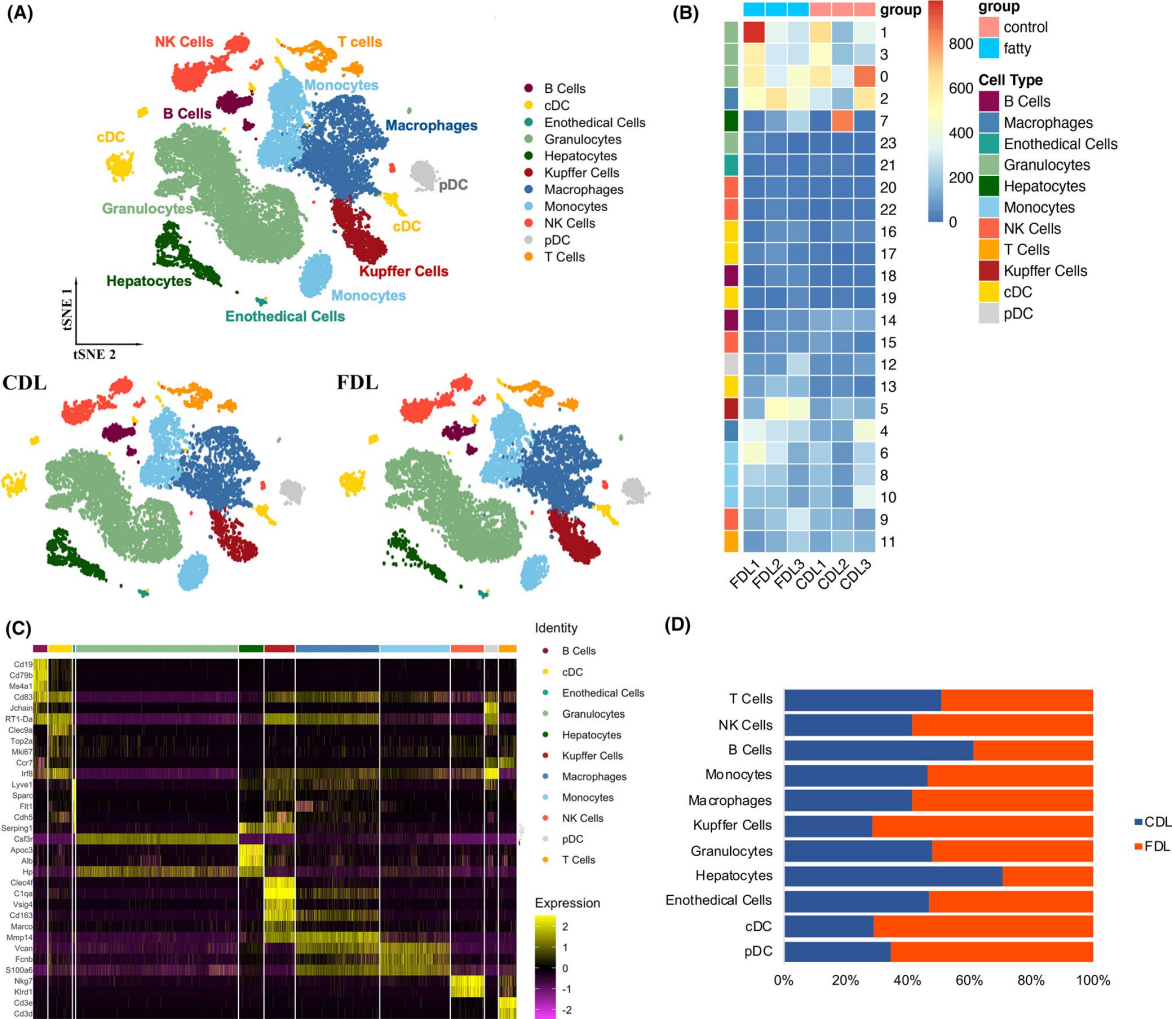
ScRNA‐seq profiling of the hepatic environment after liver transplantation. (A) T‐distributed stochastic neighbour embedding (t‐SNE) plot, embedding of jointly analysed single‐cell transcriptomes from 23 675 cells from 3 CDL and 3 FDL annotated by cell type and group (right panel). CDL, control donor liver; FDL, fatty donor liver. (B) Heatmap showing the expression of marker genes in the indicated cell types. The left bars label the specific cell types, and the number in the right corresponding to specific cell type. (C) Heatmap showing the expression of marker genes in the indicated cell types. The top colourful bars corresponding to specific cell types. (D) Histogram indicating the proportion of cells in liver tissue of each analysed sample. cDC, conventional dendritic cell; NK cells, nature killer cells; pDC, plasmacytoid dendritic cell

To construct a global transplanted liver niche atlas, we performed cell classification and marker gene identification using Seurat. 24 clusters were identified and visualized using the T‐distributed stochastic neighbour embedding (t‐SNE) method (Figure 2A,B). 23 675 single cells (both immune and non‐immune fractions) were clustered into 24 major clusters. Cluster‐specific genes were used to annotate cell types with classic markers described in previous studies: B cells (CD19^+^, CD79b^+^); DCs (IRF8^+^); granulocytes (CSF3R^+^); monocytes (CD14^+^, VCAN^+^); macrophages (CD163^+^); Kupffer (VSIG4^+^; CLEC4F^+^) cells; NK cells (NKG7^+^; KLRD1^+^); T cells (CD3e^+^; CD3d^+^); hepatocytes (Alb^+^). (Figure 2C) All these cell subtypes were shared among livers and between CDL and FDL samples, albeit at different proportions (Figure 2D). The infiltration levels of KCs, plasmacytoid DCs (pDCs) and conventional DCs (cDCs) were relatively low in CDL. Other immune cell clusters varied among samples, revealing substantial heterogeneity of immune cell compositions among transplanted livers. In short, regardless of this variability, CDL and FDL samples shared the same major immune cell subtypes and a relative similar fraction of myeloid‐ and lymphoid‐derived cells.

### 3.2 Differentiation trajectory of myeloid‐derived cells is implicated in immuno‐metabolism imbalance

**FIGURE 3:**
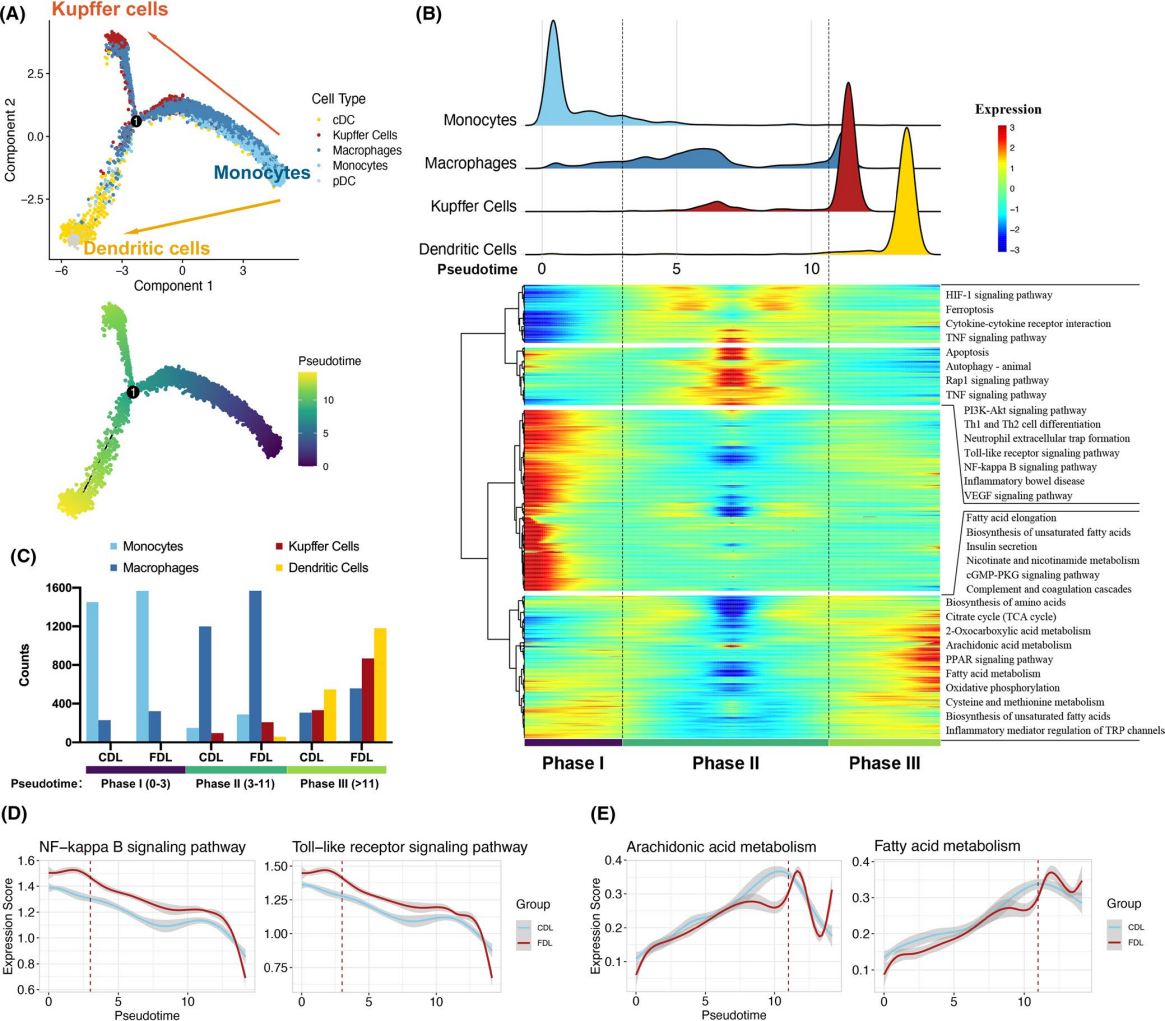
Trajectory analysis of myeloid‐derived cells. (A) Trajectory of differentiation from monocyte into macrophage and dendritic cell (DC) predicted by monocle. The lines indicating the trajectories of lineages and the arrows indicating manually added directions of the pseudotime. Dots: single cells; colours: cell types. (B) Heatmap revealing the dynamic changes in gene expression during the differentiation process. The distribution of myeloid‐derived cell subtypes during the transition was divided into 3 phases (lower panel), along with the pseudotime. Subtypes are also labelled by colours (upper panel). Difference in enriched pathways by KEGG between different phases (right panel). (C) Histogram indicating the proportion of myeloid cells in liver tissue of each group. (D‐E) Two‐dimensional plots showing the expression scores for inflammatory (D) and metabolic pathways (E), in CDL (blue) and FDL (red) samples, along with the pseudotime. CDL, control donor liver; FDL, fatty donor liver

Recent studies have highlighted the concept of functional adaptations on metabolic stimuli in myeloid‐derived immune cells during NAFLD.^23^, ^24^ We performed unsupervised clustering of myeloid cells. A total of 11 clusters emerged within the myeloid lineage, including two clusters for macrophages (Macro1 and Macro2), three for monocytes (Mono1‐Mono3), four for DCs (DC1‐DC4) and one for KCs (Figure S1). Next, we explored the dynamic immune states and cell transitions in myeloid cells by inferring the state trajectories using Monocle (Figure 3A). Pseudotime analysis showed that monocytes were at the beginning of the trajectory path, whereas the KCs and DCs at a terminal state (Figure 3A). By integrating pathways enrichment and trajectory information, this transition was determined to initiate with monocytes, through an intermediate inflammatory state mostly characterized by macrophages, and finally reached a metabolic disorder state, characterized by KCs and DCs (Figure 3B). There was a substantial heterogeneity of myeloid cell compositions between CDL and FDL along with pseudotime. Remarkably, KCs and DCs have taken the dominant position at the terminal state, with the proportion of these cells in FDL being much higher than in CDL, revealing certain kind of activation in these cells especially in FDL (Figure 3C). Through KEGG pathway analysis, the expression score of inflammatory‐related pathways including NF‐kappa B signalling pathway and Toll‐like receptor (TLR) signalling pathway is much higher in FDL group at intermediate phase (Figure 3D). FDL group at terminal phase, nevertheless, was capable of a metabolic disorder phenotype, demonstrated by a trend of higher expression score of arachidonic acid metabolism and fatty acid metabolism (Figure 3E).

### 3.3 An inflammation‐associated Kupffer cell subtype was identified in FDL

**FIGURE 4:**
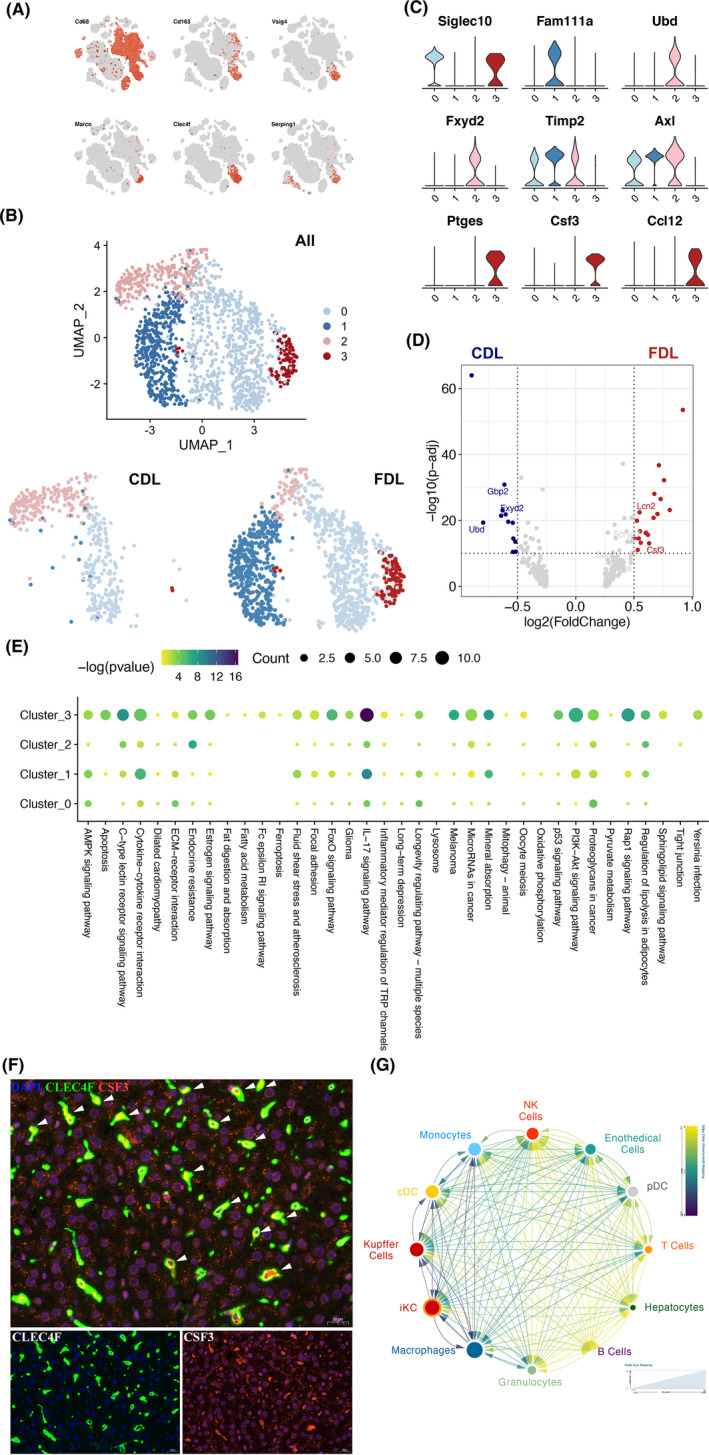
Transcriptome heterogeneity of different subsets of Kupffer cells. (A) t‐SNE plots of the expression of representative marker genes for Kupffer cells. (B) UMAP plot of Kupffer cells by clusters (up) and group (down). CDL, control donor liver; FDL, fatty donor liver. (C) Violin plot showing the expression of marker genes in the indicated clusters of Kupffer cells. The colourful bars corresponding to specific clusters in (b). (D) Volcano plot shows differentially expressed genes between CDL (blue dots) and FDL Kupffer cells (red dots). The names of some significant genes are indicated in the plots. (E) The KEGG pathway enrichment analysis of differentially expressed genes (DEGs) among Kupffer cell subtypes. (F) Multiplexed immunofluorescence staining to validate the existence of CSF3^+^ Kupffer cells (KCs) in FDL sample, white arrows (CLEC4F^+^CSF3^+^). Scale bar, 20 μm. (G) Connection graph showing the intensity of interactions between one cell type to another in coloured circles. Interactions were evaluated among all cell types, including immune and non‐immune fractions. The node size indicated the number of L‐R interactions

We next examined a small number of known biomarkers for KCs^25^, ^26^, ^27^ and discovered that all of these genes were differentially expressed in this cell type (Figure 4A). After that, KCs were clustered into four different subtypes (Figure 4B), and the exclusive marker genes for each subtype were shown in Figure 4C. Thereinto, cluster_3 KCs was identified as CSF3^+^ KCs, also renamed as inflammation‐associated Kupffer cell subtype (iKC), which exhibited a strong expression of various cytokines and chemokines, including CSF3 and CCL12. To investigate the function of each subtype, we performed GO enrichment analysis on the DEGs between CDL and FDL. As shown in Figure 4D, adipocyte‐related gene, LCN2, was also significantly up‐regulated in FDL. Through the activation/infiltration of intragraft macrophages after liver transplantation, LCN2 has been previously characterized as an inflammatory meditator and implicated in fatty liver graft injury.^28^ KEGG pathway analysis revealed that iKC was associated with IL‐17 signalling pathway, cytokine‐cytokine receptor interaction pathway, PI3K‐AKT signalling pathway and Rap1 signalling pathway (Figure 4E). The results demonstrated that iKC highly contributed to a pro‐inflammation status, playing a pivotal role in fatty liver graft injury. Since the IL‐17 signalling pathway was mostly enriched in iKC, we investigated the expression level of top 10 marker genes (Figure S2). Multiplexed immunofluorescent staining was performed to provide evidence that iKC was increased in FDL group (Figure 4F). In addition, we added two extra control groups to demonstrate and compare the iKC in rat livers with immunofluorescent both before and after transplantation (Figure S3). To further investigate the interactions that occur in the ecosystem, we calculate the interactions strength using significant L‐R pairs. Connection graph shows the strong intensity of interactions between iKC to other cell types (Figure 4G). Our results demonstrated that iKC in the FDL exhibited enhanced pro‐inflammatory capability in fatty liver graft injury.

### 3.4 Distinct clusters and gene expression of DCs were identified in steatotic transplanted liver

**FIGURE 5:**
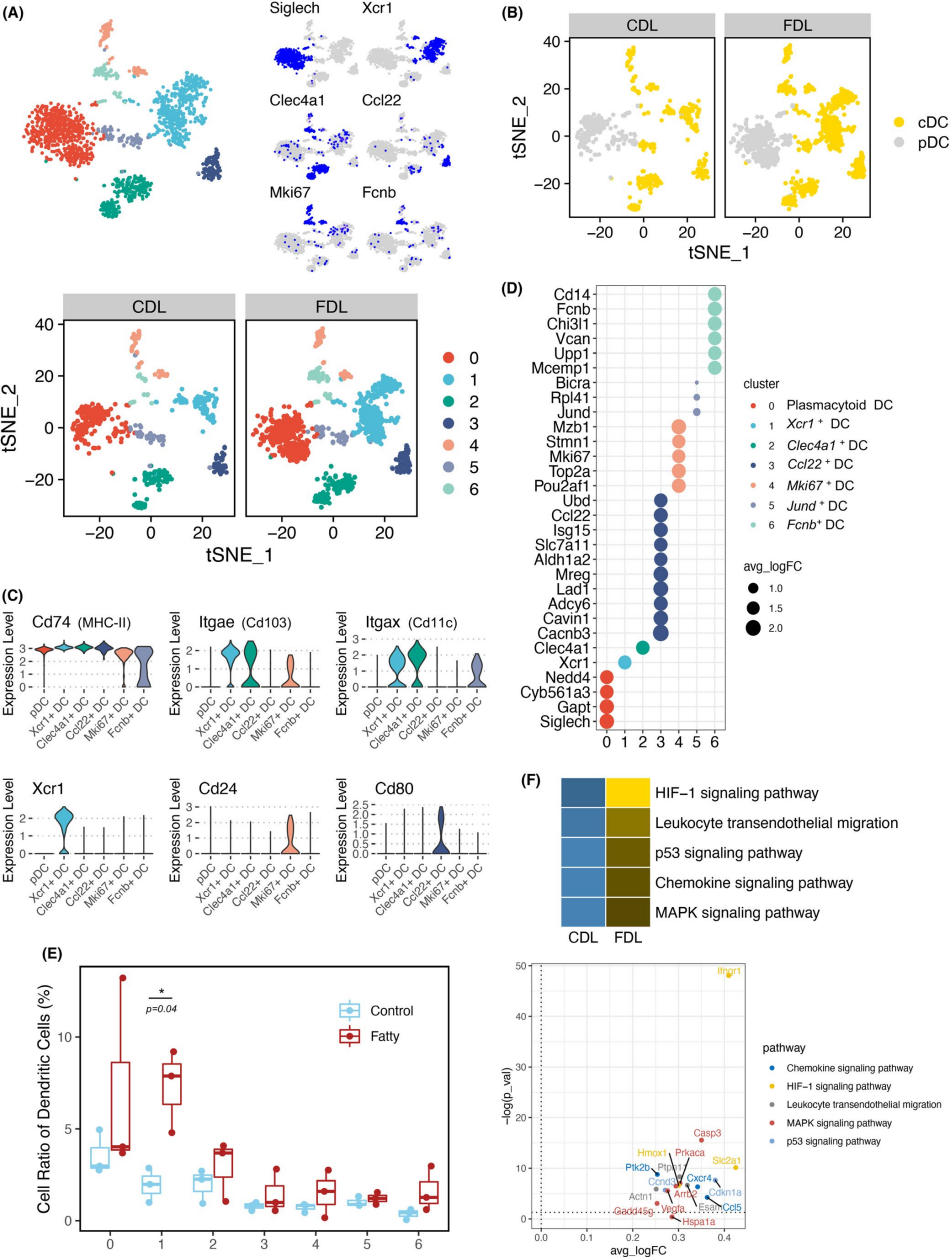
Distinct subsets of DCs identified in FDL after liver transplantation. (A) t‐SNE plot of DCs by clusters (up) and group (down). Expression patterns of selected markers for the identification of DC subsets were also shown in top right corner. Blue indicates high expression and grey indicates low or no expression. (B) t‐SNE plot of DCs in different groups coloured by pDC (grey) and cDC (yellow). (C) Violin plot showing the expression of marker genes in all subsets of DCs. The colourful bars corresponding to specific clusters in (a). (D) Dot plots showing the expression of marker genes in each subset of DCs. (E) Boxplot showing the fraction of DC subsets in CDL (blue) and FDL (red). (F) Significant KEGG pathways enriched by up‐regulated DEGs between CDL and FDL in XCR1^+^ DCs were shown in heatmap (up) and some significant genes are indicated in volcano plot (down). DEGs, differentially expressed genes. CDL, control donor liver; DCs, dendritic cells; FDL, fatty donor liver

Based on function, phenotype and tissue distribution, DCs can be divided into two major subtypes: pDCs and cDCs. The re‐clustering of DCs revealed seven distinct subsets and cluster_1‐6 DCs were classified into cDCs (Figure 5A,B). All these subsets were shared across CDL and FDL samples. Transplanted liver‐infiltrated cDCs had unique transcriptional profiles compared with pDCs. We noticed that CD103 was extremely enriched in cDCs (Figure 5C), suggesting their regulatory role to influence the inflammatory milieu of the fatty liver.^29^ In addition, we identified each subset's feature according to the expression of marker genes in each subset of DCs (Figure 5D). DC subsets showed variation among groups, ranging from 0.1% to 13% of the total immune fraction. The proportion of cluster_1 DCs with high expression of XCR1 (XCR1^+^ DCs) was significantly higher in FDL than in CDL samples (*P* = .04) (Figure 5E). By performing GO enrichment analysis on the DEGs between CDL and FDL, we found that XCR1^+^ DCs were related to HIF‐1 signalling pathway, leucocyte transendothelial migration, chemokine signalling pathway and MAPK signalling pathway (Figure 5F), indicating a potential role in activating cellular signalling to exacerbate the hepatic I/R injury.^30^, ^31^, ^32^ In conclusion, DCs in FDL group characterized by overexpression of XCR1 demonstrated an efficient antigen‐presenting ability and a crucial role in linking the innate and adaptive immune response.

### 3.5 Cross‐presenting XCR1^+^ DCs as targets to prime CD8^+^ T cells response

**FIGURE 6:**
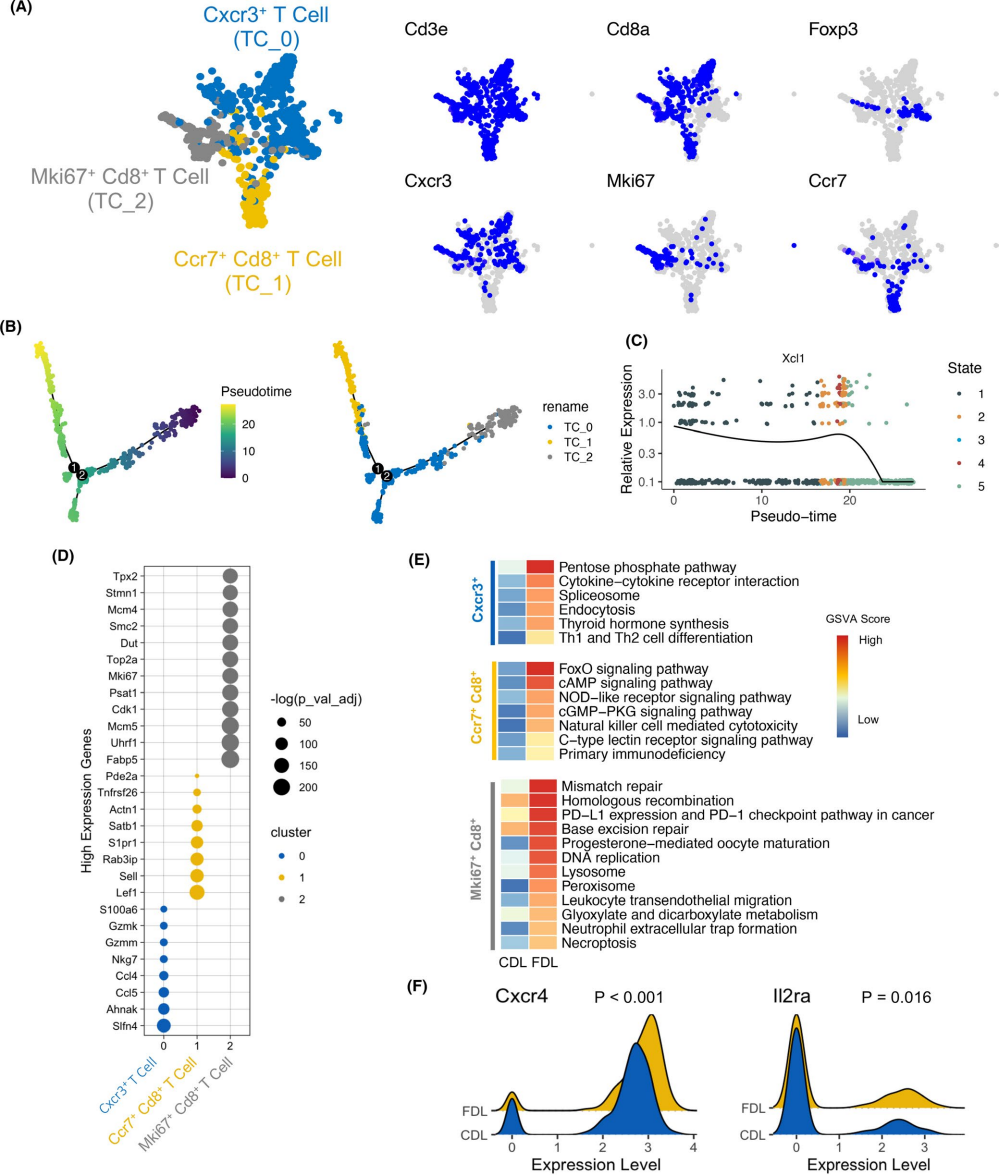
Characteristics and relationships of T‐cell subsets. (A) t‐SNE plot of T cells by clusters (left) and expression patterns of selected markers (right). Blue indicates high expression and grey indicates low or no expression. (B) Trajectory of all clusters of T cell along pseudotime in a two‐dimensional state‐space defined by Monocle2. Each point corresponds to a single cell, and each colour represents a T‐cell cluster. (C) Two‐dimensional plot showing the dynamic expression of XCL1 during the T‐cell transitions along the pseudotime. (D) Dot plots showing the expression of marker genes in each subset of T cells. (E) Significant KEGG pathways enriched by up‐regulated DEGs between CDL and FDL in each subset of T cells. (F) The expression of CXCR4 and IL2RAwas indicated in ridge plot. CDL, control donor liver; FDL, fatty donor liver

It is reported that the XCL1‐XCR1 axis plays an important role in DC‐mediated cytotoxic immune response.^33^ To further investigate the unique subtype of T cells corresponding to XCR1^+^ DCs, we determined three T‐cell phenotypes (CXCR3^+^ T cell, CCR7^+^ CD8^+^ T cell and Mki67^+^ CD8^+^ T cell) by re‐clustering (Figure 6A). Then, the dynamic immune states and cell transitions in liver‐infiltrated T cells were explored by inferring the state trajectories using Monocle. This analysis showed that the Mki67^+^ CD8^+^ T cells were present at the beginning of the trajectory path, whereas the CCR7^+^ CD8^+^ T cell was at a terminal state (Figure 6B). According to the comparative expression analysis in different subtypes of T cells (Figure S4), we observed that XCL1 was highly expressed in Mki67^+^ CD8^+^ T cell and CXCR3^+^ CD8^+^ T cell along with pseudotime, whereas significantly reduced in CCR7^+^ CD8^+^ T cell (Figure 6C). This phenomenon revealed that CD8^+^ T cells underwent a transition from activation to function during FDL liver transplantation.

Differentially expressed gene (DEG) analysis indicated the top up‐regulated genes in three subtypes of T cells (Figure 6D). KEGG analysis combined with GSVA score showed that CCR7^+^ CD8^+^ T cell in FDL was characterized by up‐regulated FoxO signalling pathway and NOD‐like receptor pathway (Figure 6E), suggesting a pro‐apoptotic and pro‐inflammatory effect.^34^, ^35^ CXCR4 was reported to exaggerate the I/R injury due to enhanced recruitment of inflammatory cells, increased TNF‐α production and activation of cell death/apoptotic pathways.^36^ In accordance with this study, we found that CXCR4 was significant higher in T cells of FDL group, imprinting that CXCR4 was also involved in the fatty liver graft injury after liver transplantation (Figure 6F). We also noted that IL2 receptor subunit alpha (IL2RA) was predominant in FDL group (Figure 6F). IL2RA and other two chains, IL2RB and IL2RG, constitute the high‐affinity IL2 receptor. Considering IL2 receptor antagonist‐based induction therapy was effective in reduction of acute rejection after liver transplantation,^37^ treatment for acute rejection in patients underwent fatty liver transplantation might focus on IL2 receptor early.

### 3.6 Constructing an XCR1^+^ DC‐based regulatory network for fatty liver graft injury

**FIGURE 7:**
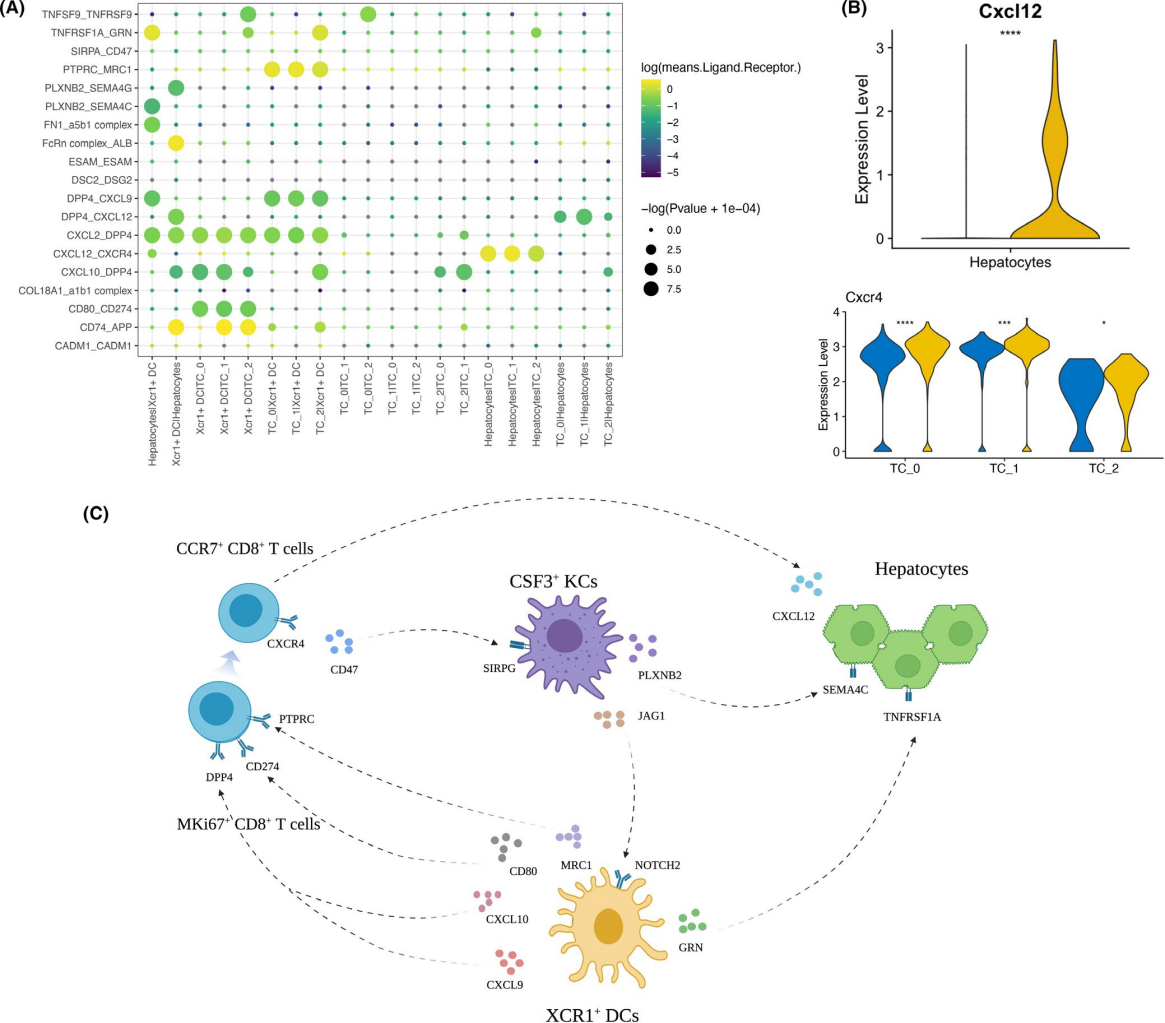
Cell‐cell communication network in FDL after liver transplantation. (A) Bubble plots show ligand‐receptor (L‐R) pairs among XCR1^+^ DCs, T cells and hepatocytes. (B) Violin plot showing the expression of selected ligands (up) and receptors (down) in CDL and FDL, based on the expression of indicated cell types. *P* values were calculated by Student's *t* test. (C) Predicted regulatory network centred on XCR1^+^ DCs and CSF3^+^ KCs. CDL, control donor liver; DCs, dendritic cells; FDL, fatty donor liver; KCs, Kupffer cells

Using Cellphone DB, we investigated the cell‐cell interaction network among the cell types identified in our present work. XCR1^+^ DC showed the most interactions with T cells and hepatocytes (Figure 7A). Considering the results of GO analysis and GSEA and the expression abundance in our data, secretion of CXCL9 and CXCL10 by XCR1^+^ DC was responsible for the immune infiltration status of FDL. DPP4, as the receptor of these ligands, is widely expressed on other immune cells. Moreover, MRC1 secreted by XCR1^+^ DC could interact with PTPRC, which is highly expressed on a subtype of CD8^+^ T cells. MRC1 was previously reported to play a critical role in myeloid plasticity, which in turn affects the adaptive immune response.^38^ Interaction between programmed death ligand‐1 (PD‐L1, CD274) and the immunostimulatory molecule CD80 was also found in this regulatory network and functioned as a checkpoint to regulate immune responses. The interactions of PD‐L1 with CD80 augment CD8^+^ T cell expansion without increasing anergy, exhaustion or apoptosis, thus exacerbating the CD8^+^ T‐cell immune response, would eventually lead to the severity of graft‐versus‐host disease (GVHD).^39^ Taken together, our results predicted that XCR1^+^ DC could promote the proliferation of T cells and potentially be able to recruit CD8^+^ T cells into the microenvironment.

Notably, we found that T cells showed the strongest chemokine interactions with hepatocytes in FDL group via CXCR4/CXCL12 axis, mediating the directional migration of CXCR4‐expressing T cells to CXCL12‐expressing hepatocytes (Figure 7B). It was previously reported that signalling through CXCR4 is detrimental to liver recovery and regeneration,^40^ and we pinpointed their origin to T cells. Intriguingly, we also found that a XCR1^+^ DC‐hepatocytes interaction, namely GRN/TNFRSF1A axis, was up‐regulated in FDL group. The GRN gene encodes a protein called progranulin (PGRN), which is highly expressed in macrophage and monocyte‐derived DCs.^41^ PGRN regulates inflammatory responses by counteracting tumour necrosis factor (TNF)‐mediated inflammatory signalling pathway.^42^

In a nutshell, cells from FDL group after LT could compromise antigen presentation in XCR1^+^ DC cells via the CXCL9‐DPP4, CXCL10‐DPP4, MRC1‐PTPRC and CD80‐CD274 axes, recruit T cells and thus interact with hepatocytes via the CXCR4/CXCL12 axis, which altogether exacerbate the transplanted liver injury through the activation of CD8^+^ T cells. Furthermore, we also analyse the connection of CSF3^+^ KCs with DCs, CD8^+^ T cells or hepatocytes. iKCs in the FDL not only exert an effect on hepatocytes directly through PLXNB2/SEMA4C axis but also influence the antigen presentation and adaptive immune response via JAG1/NOTCH2 and CD47/SIRPG axes, respectively (Figure S5). Thus, iKCs in the FDL exhibit an enhanced pro‐inflammatory capability in fatty liver graft injury. In summary, our results demonstrated that reciprocally interacting signalling pathways control transplanted liver injury in an FDL‐specific manner (Figure 7C).

## 4 DISCUSSION

With a rising number of new registrants in transplant waitlists, the disparity between allograft availability and waiting candidates remains persistent. To bridge this gap, there has been increasing support for the use of steatotic livers.^7^, ^43^ However, accumulating evidences showed that using steatotic grafts for LT is associated with higher risks of complications after LT.^44^ The underlying mechanisms of the increased susceptibility of steatotic livers to I/R injury were still uncertain. Here, we generated a single‐cell transcriptome atlas and revealed the components of the microenvironment inside transplanted livers.

In the present study, we identified 11 different cell types in the transplanted liver microenvironment. A high degree of heterogeneity in the distribution, functional properties, transcriptional regulation and cell‐cell interactions of different immune subsets in liver transplantation using steatotic grafts were unveiled. We have also identified two new subsets namely CSF3^+^ KCs, a pro‐inflammatory cluster enriched in FDL group, and XCR1^+^ DCs, related to the recruitment of CCR7^+^ CD8^+^ T cells in transplanted steatotic liver microenvironment.

The unique inflammatory polarization in myeloid cells induced by NAFLD progression would alter the response to injury signals.^23^ Therefore, we re‐analysed the scRNA‐Seq data of myeloid cells and divided them into four cell types and 11 clusters. By integrated pathways enrichment analysis and trajectory information, a transition from ‘inflammatory state’ to ‘metabolic disorder state’ was identified in the milieu of myeloid cells. Then, we focussed on the metabolic remodelling in terminal state by analysing KCs and DCs specifically.

The role of KCs in I/R injury after LT might be self‐limiting. They not only were involved in the development of the pro‐inflammatory response in I/R injury, but also functioned to terminate this reaction.^45^ In our work, we observed that a subset of KCs, CSF3^+^ KCs, contributed abundantly to a pro‐inflammation milieu. CSF3^+^ KCs might be the pivotal subset of KCs in the initiation of pro‐inflammatory response in steatotic I/R injury. Yang et al^46^ reported that IL‐17a promoted graft injury in rat orthotopic LT with fatty graft and also mechanically elucidated that IL‐17a exacerbated the fatty liver I/R injury through the increased neutrophil infiltration and mitochondria‐driven apoptosis. Consistent with Yang's study, we found that the IL‐17 signalling pathway was significantly enriched in FDL group and we pinpointed their origin to CSF3^+^ KCs. In a word, we identified the IL‐17 signalling in CSF3^+^ KCs as a potential regulatory axis of cellular response to hepatic I/R injury and this result warrants further studies in human patients. It is worth noting that LCN2 was one of the top ten up‐regulated genes involved in IL‐17 signalling pathway. LCN2, also known as neutrophil gelatinase‐associated lipocalin (NGAL), is released by various cell types and is an attractive biomarker of inflammation, ischaemia and infection.^47^ Cheng et al^28^ reported that LCN2 aggravated small‐for‐size fatty liver graft injury through upregulation of cytokines and promotion of macrophage infiltration in a rat orthotopic LT model. Moreover, LCN2 was further validated in clinical samples after living‐donor liver transplantation (LDLT). In our study, LCN2 was also significantly up‐regulated in FDL group, especially in KCs, and involved in many inflammation‐related pathways. LCN2 was capable of much potential in acting not only as a biomarker of hepatic injury in fatty graft after liver transplantation but also as a therapeutic target to prevent fatty liver graft injury in LT.

On the other hand, DCs excel at antigen presentation and play a vital role in regulating both innate and adaptive immune responses. Endogenous danger signals, damage‐associated molecular patterns (DAMPs) and pathogen‐associated molecular patterns (PAMPs), can be recognized by pattern recognition receptors (PRRs) during the process in innate immune system, thus triggering consequently inflammatory responses.^48^ DCs express a large repertoire of PRRs, including TLRs and C‐type lectins that can recognize signals by profound phenotypic and functional change.^49^ After interaction with DAMPs and PAMPs, DCs secrete pro‐inflammatory cytokines, chemokines, and other mediators. A previous study documented that increasing DC numbers expressing functional TLR4 are critical to the full manifestation of hepatic I/R injury. DCs are likely to be one of the initial responders to the release of DAMPs from damaged or necrotic cells with subsequent signalling and activation of TLR4.^50^ Another study suggested that recruited blood‐borne DCs mainly displayed pro‐inflammatory activities in a sterile microenvironment.^51^ Intriguingly, we noticed that the number of DCs, especially the cDC part, was higher in FDL than CDL group (Figure S6), indicating that cDCs exert a stronger pro‐inflammatory response in the transplanted steatotic liver microenvironment.

In addition, DCs are known as antigen‐presenting cells (APC) to initiate adaptive immune responses. Activated DCs express high levels of major histocompatibility complex (MHC) molecules on their surface and present pathogen‐derived antigens to bearing to potently activate antigen‐specific T cells.^52^ In this study, we have identified XCR1^+^ DCs as a distinctive subset of DCs in FDL group. It was reported that XCL1 is mainly produced by activated CD8^+^ T cells and considered to be the only ligand of receptor XCR1.^33^ However, the role of cytotoxic CD8^+^ T lymphocytes that orchestrate immune responses against viruses and tumours remains unclear in the context of I/R injury. Recently, emerging researches in acute tissue injury are highlighting the importance of cytotoxic CD8^+^ T during the I/R injury. A study showed that cytotoxic CD8^+^ T lymphocytes were recruited in the ischaemic heart following acute myocardial infarction (MI) and foster cardiomyocyte death through the local release of Granzyme B, leading to increased myocardial inflammation, tissue injury and deterioration of myocardial function.^53^ Intriguingly, the Deutsches Krebsforschungszentrum (DKFZ) recently reported that the progressive accumulation of exhausted, unconventionally activated CD8^+^ PD1^+^ T cells in non‐alcoholic steatohepatitis (NASH)‐affected livers were enriched in pathways involved in inflammatory signalling and NK cell‐like cytotoxicity was correlated with liver damage.^54^ At the same time, Dudek, M. et al^55^ revealed that liver‐resident CXCR6^+^ CD8^+^ T cells are rendered auto‐aggressive through defined, non‐redundant sequential activation steps in immune‐mediated metabolic diseases such as NASH, causing a chronic liver damage. They found that the killing mediated by auto‐aggressive CD8^+^ T cells was mechanistically different from that by antigen‐specific cells. Also, Kolachala et al^56^ reported that CD8^+^ T cell was an important cell extrinsic component mediating the severer liver damage by I/R injury. By blocking CD8^+^ T cells, either from inhibiting trafficking or from depleting could protect against I/R injury in a steatotic liver. Partially consistent with these studies, our data showed that XCL1 was highly expressed in Mki67^+^ CD8^+^ T cell and CXCR3^+^ CD8^+^ T cell, characterized as a possible ‘activation’ status in FDL. Although there is no significance between two groups after calculating the T‐cell subtypes cell ratio between the two groups (Figure S7), we could find a tendency that more TC_1 cells (CCR7^+^ CD8^+^ T cells) were accumulated in FDL group. The discrepancy in the group was possibly caused by limited samples. Even so, KEGG analysis combined with GSVA score using the top up‐regulated genes in three subtypes of T cells indicated that the terminal state of T cells, CCR7^+^ CD8^+^ T cells in FDL, was characterized as more ‘function’ as they exerted pro‐apoptotic and pro‐inflammatory effects in transplanted steatotic liver microenvironment. Taken together, our findings highlight the importance of CD8^+^ T cells transition in I/R injury in a steatotic liver, indicating that interventions on them might be effective in settings of hepatic I/R injury to minimize liver damage.

In addition to these notable findings, our study should be interpreted in light of several limitations. Firstly, a study indicated that the different liver steatosis models possessed distinct characteristics and showed different response against ischaemia reperfusion injury after transplantation.^57^ However, the degree of steatosis in our models was validated in various aspects to minimize this discrepancy. Secondly, although our research revealed expression of many novel cell‐type‐specific genes in transplanted steatotic liver, the limited number of cells, around 4000 cells on average for each sample, might hinder the identification of rare cell types and thus underestimate the heterogeneity of intercell interactions. Thirdly, due to the shortage of antibodies to target rat cell surface markers, validation and quantification of XCR1^+^ DC cells seem to be difficult. We have attempted to perform the IHC experiment of XCR1 using a theoretically usable antibody but the preliminary tests indicated that this antibody did not work in IHC for rat liver tissue (Figure S8). Moreover, the distinctive cell subsets identified in our study were not validated in samples from LT patients, which is mostly due to the difficulty in obtaining transplanted livers in clinical.

In short, our results firstly delineate the distinct immune subsets and their underlying transcriptome dynamics in regulation of hepatic I/R injury in fatty liver. Also, this comprehensive analysis highlights the complex cell‐cell crosstalks that occur during the liver transplantation using steatotic grafts. Additionally, our datasets, as provided in an interactive portal (https://ciopm.shinyapps.io/scrna‐flgilt/), can be used as a resource for facilitating a deeper understanding of the mechanisms associated with hepatic I/R injury in fatty liver and assisting in developing novel therapeutic strategies to benefit more LT patients.

## CONFLICT OF INTEREST

All authors have no financial disclosures or conflicts of interest to declare.

## AUTHOR CONTRIBUTIONS

XX conceived and supervised the project. XX, XY, DL and RW designed the experiments. XY and ZL performed the experiments and conducted all the sample preparation for scRNA‐Seq with help from ZL, JZ, XW and QW; XY and RW performed the data analysis with help from MY and HC; XY and RW wrote the manuscript with the help from XX, WT and all the other authors.

## ETHICAL APPROVAL

The animal experiments were conducted according to the protocol approved by the Zhejiang University after the review by the Institutional Animal Care and Use Committee.

## Supporting information

Figure S1‐S8

Table S1

Table S2

## Data Availability

The raw single‐cell sequence data reported in this paper have been deposited in the Genome Sequence Archive in National Genomics Data Center,^58^, ^59^ China National Center for Bioinformation/Beijing Institute of Genomics, Chinese Academy of Sciences, under accession number CRA004061 that are publicly accessible at https://bigd.big.ac.cn/gsa.
