# Supplementary material for: Single‐cell profiling reveals distinct immune phenotypes that contribute to ischaemia‐reperfusion injury after steatotic liver transplantation
Source: Cell Prolif. 2021 Sep 1;54(10):e13116. doi: 10.1111/cpr.13116 (PMC8488562; doi:10.1111/cpr.13116)

**Supplementary Tables and Figures**

**Supplementary Table S1. Cell type defined by known marker genes.**

**Supplementary Table S2. A summary of quality control results.**

**Figure S1.** Heatmap showing the subtypes of myeloid-derived cells derived from CDL and FDL samples.

**Figure S2.** UMAP plots of Kupffer cells, indicating the expression of top marker genes involved in IL-17 signaling pathway.

**Figure S3.** Multiplexed immunofluorescence staining to demonstrate and compare the existence of CSF3^+^ KCs both before and after transplantation. Scale bar, 20 μm.

**Figure S4.** Violin plot showing the expression of XCL1 in each subset of T cells.

**Figure S5.** Bubble plots show ligand-receptor (L-R) pairs among CSF3^+^ KCs, DCs, T cells and hepatocytes.

**Figure S6.** The comparison of cDCs counts between CDL and FDL group.

**Figure S7.** The comparison of T cell ratio between CDL and FDL group in three T cell subtypes.

**Figure S8.** IHC staining of XCR1 using a theoretically usable antibody (BioLegend, #148202) in a FDL sample. (x100)

**Figure S1.**


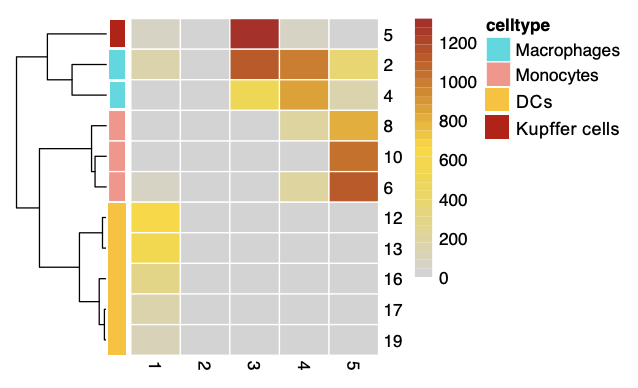


**Figure S2.**

**Figure S3.**

**
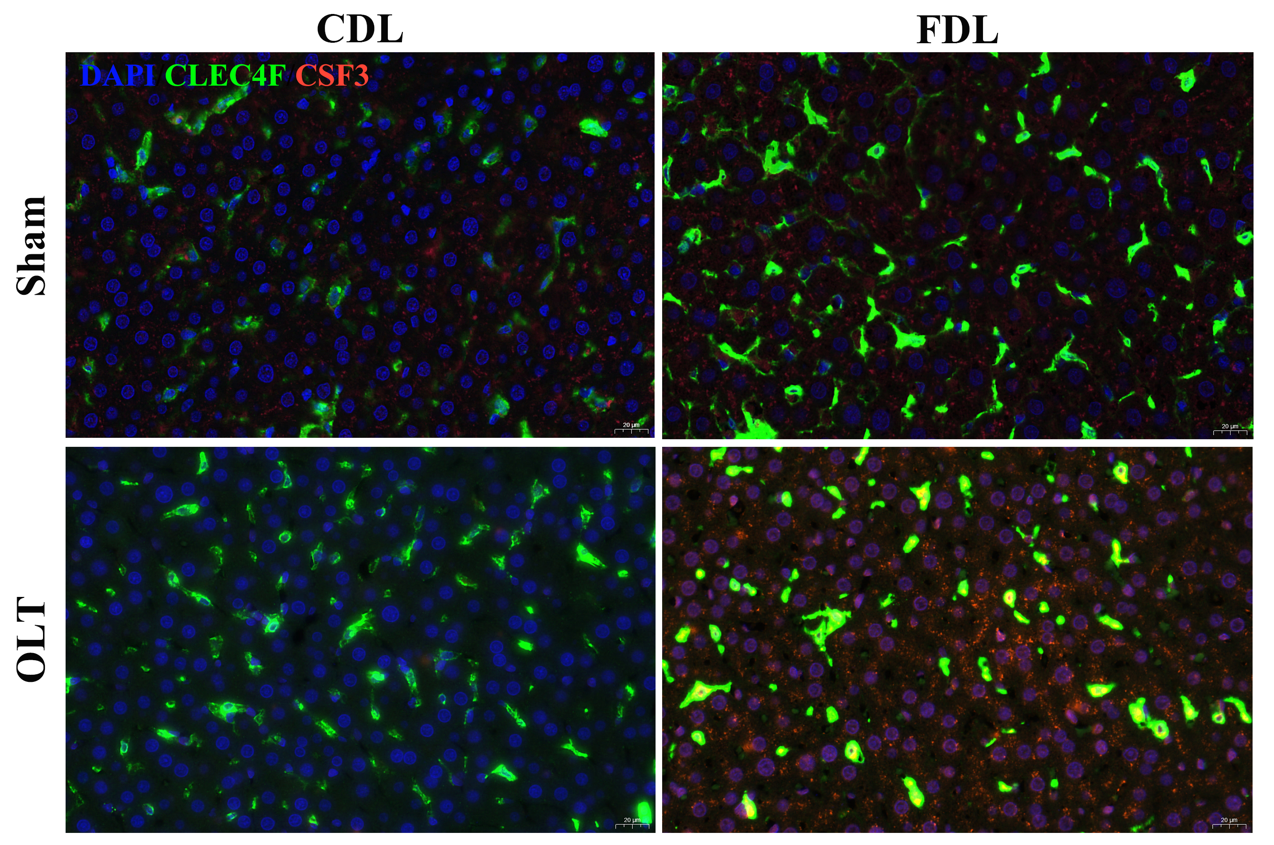
**

**Figure S4.**


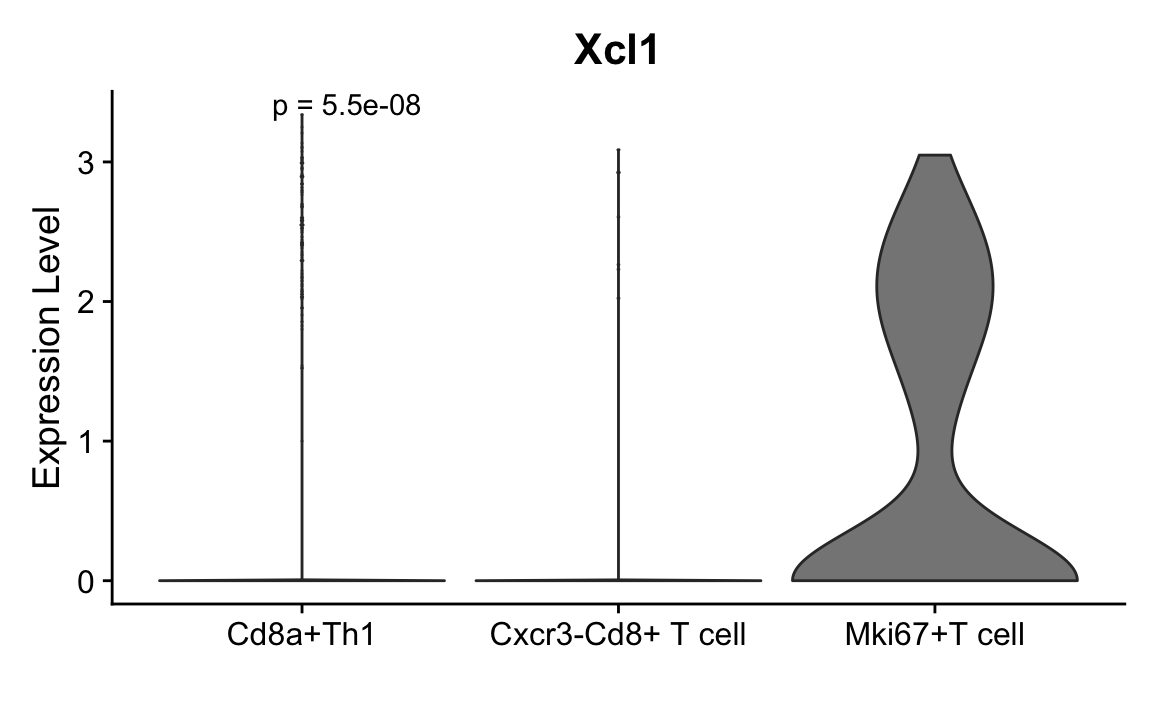


**Figure S5.**

**
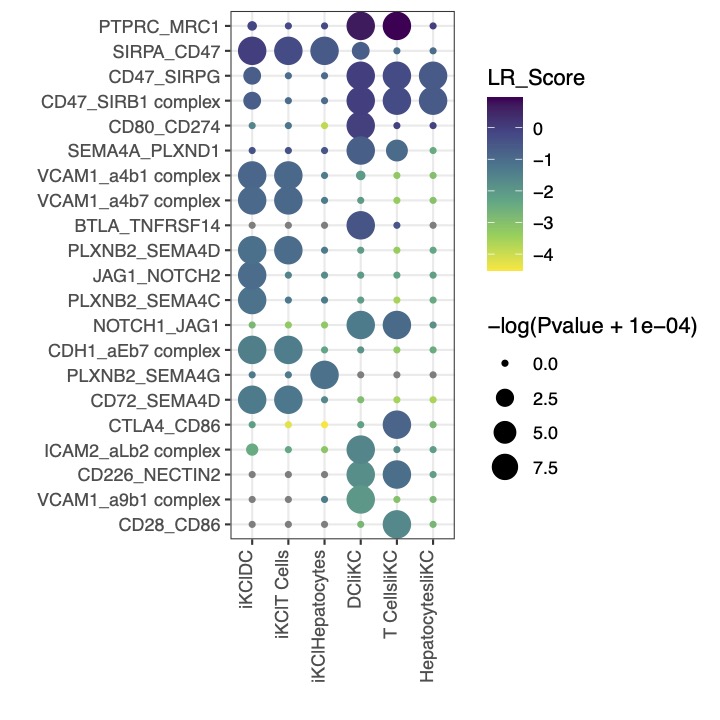
**

**Figure S6.**


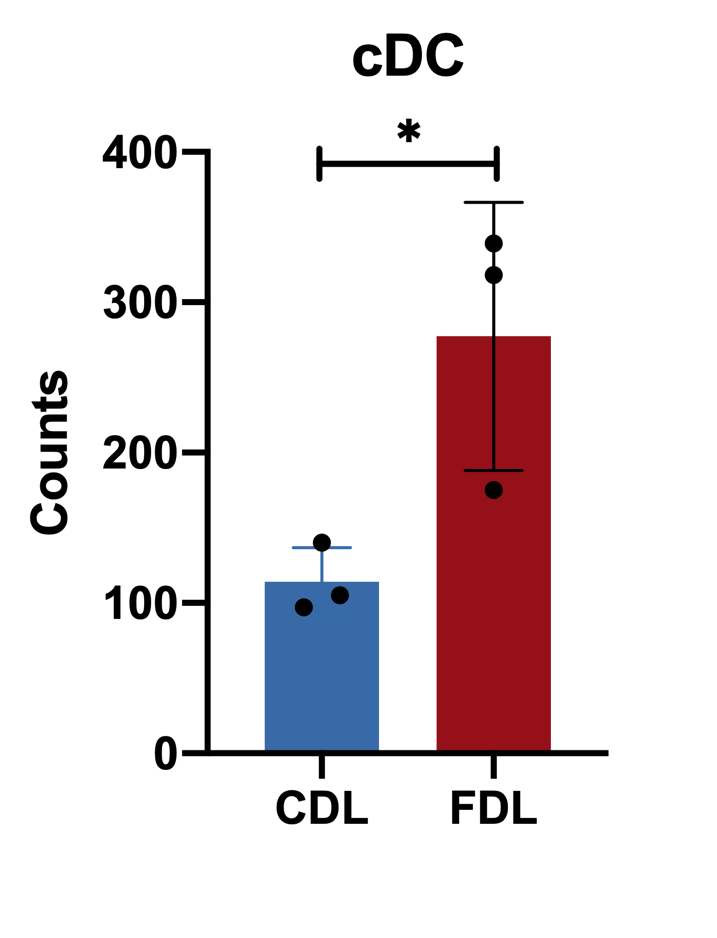


**Figure S7.**


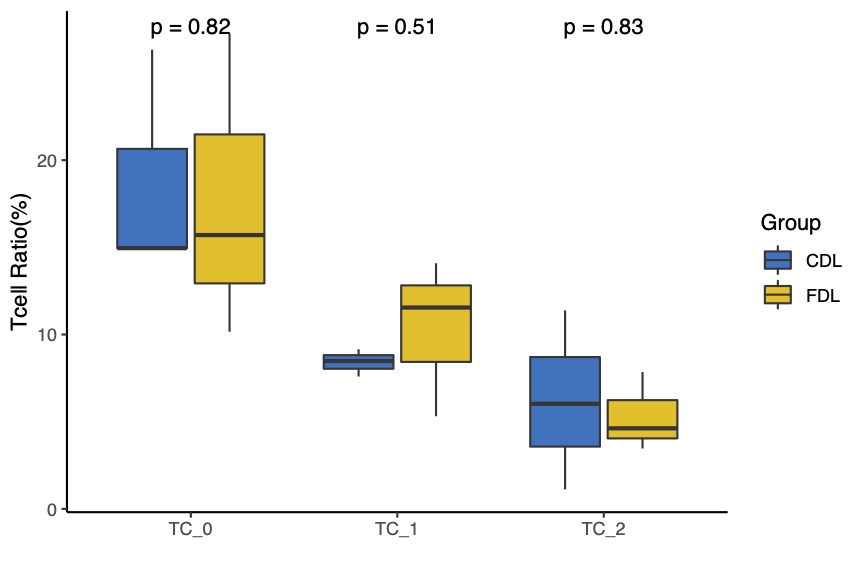


**Figure S8.**


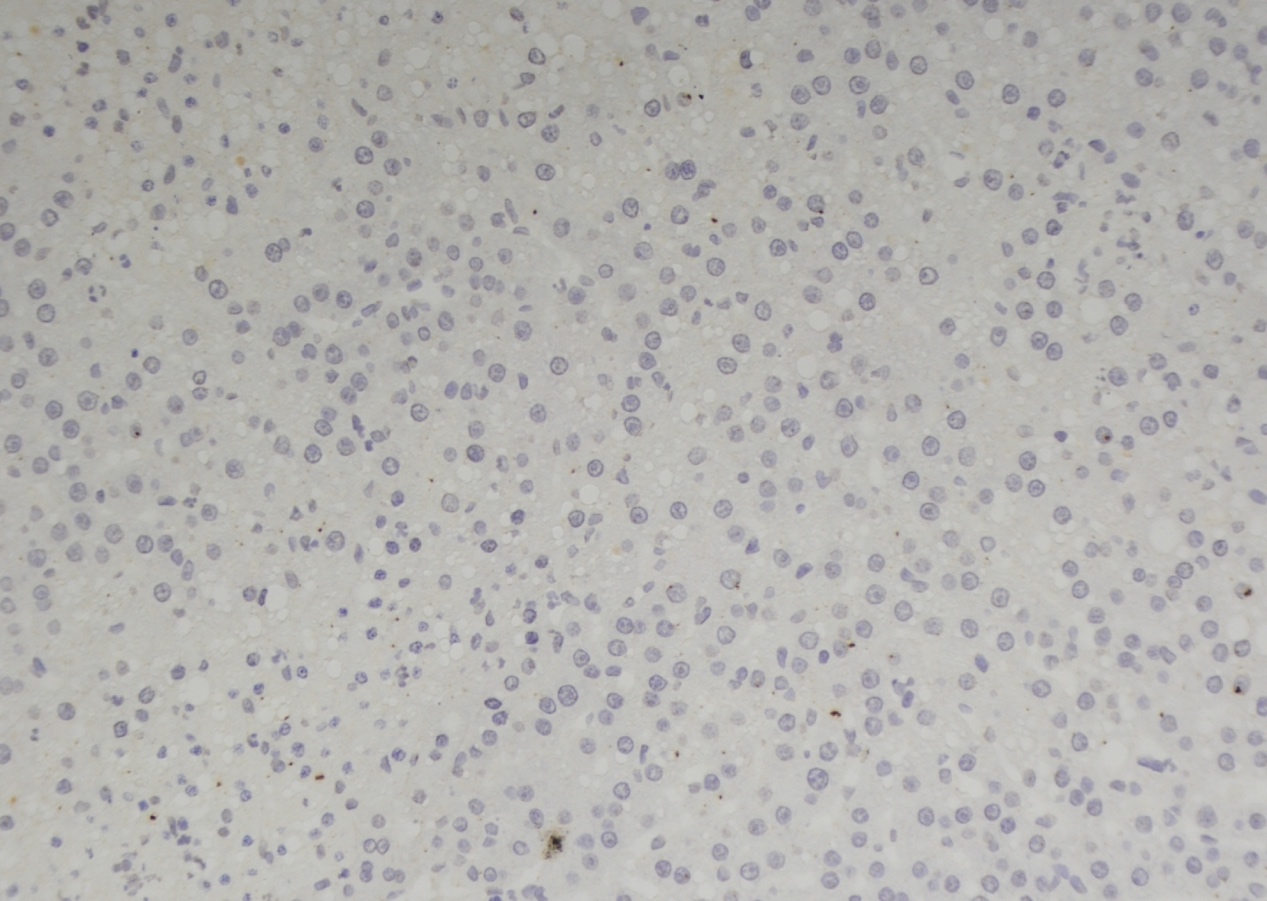

Supplement: Supplementary file 1 — Figure S1‐S8 [file CPR-54-e13116-s003.docx]
