# Supplementary material for: Single‐cell profiling reveals distinct immune phenotypes that contribute to ischaemia‐reperfusion injury after steatotic liver transplantation
Source: Cell Prolif. 2021 Sep 1;54(10):e13116. doi: 10.1111/cpr.13116 (PMC8488562; doi:10.1111/cpr.13116)
Supplement: Supplementary file 3 — Table S2 [file CPR-54-e13116-s002.docx]

**Supplementary Table S2. A summary of quality control results.**

| **Sample** | **CDL_1** | **CDL_2** | **CDL_3** | **FDL_1** | **FDL_2** | **FDL_3** |
| --- | --- | --- | --- | --- | --- | --- |
| before_filtering_total_reads | 689640514 | 688322794 | 981688208 | 5.43E+08 | 6.02E+08 | 6.62E+08 |
| before_filtering_total_bases | 1.03446E+11 | 1.03248E+11 | 1.47253E+11 | 8.14E+10 | 9.04E+10 | 9.93E+10 |
| before_filtering_q20_bases | 95884775601 | 95494647896 | 1.33464E+11 | 7.47E+10 | 8.26E+10 | 9.09E+10 |
| before_filtering_q30_bases | 88364406917 | 87753694081 | 1.21225E+11 | 6.85E+10 | 7.55E+10 | 8.34E+10 |
| before_filtering_q20_rate | 0.926905865 | 0.924901792 | 0.906357938 | 0.917572 | 0.914422 | 0.916075 |
| before_filtering_q30_rate | 0.854207423 | 0.849927726 | 0.823239803 | 0.841854 | 0.836119 | 0.839883 |
| before_filtering_gc_content | 0.379756 | 0.384133 | 0.383027 | 0.379612 | 0.373307 | 0.370834 |
| after_filtering_total_reads | 657624682 | 651191512 | 934220774 | 5.13E+08 | 5.63E+08 | 6.14E+08 |
| after_filtering_total_bases | 96443547021 | 95345425041 | 1.37195E+11 | 7.49E+10 | 8.19E+10 | 8.92E+10 |
| after_filtering_q20_bases | 89810393263 | 88665342527 | 1.25147E+11 | 6.9E+10 | 7.53E+10 | 8.23E+10 |
| after_filtering_q30_bases | 83107324205 | 81850063285 | 1.14228E+11 | 6.36E+10 | 6.92E+10 | 7.58E+10 |
| after_filtering_q20_rate | 0.93122242 | 0.929938091 | 0.912183852 | 0.921913 | 0.91978 | 0.921837 |
| after_filtering_q30_rate | 0.861719905 | 0.858458214 | 0.832598361 | 0.849298 | 0.845247 | 0.84969 |
| after_filtering_gc_content | 0.392103 | 0.39706 | 0.395869 | 0.394296 | 0.391288 | 0.39051 |
| ReadsFilter% | 95.35760569 | 94.60554229 | 95.16471385 | 94.55917 | 93.41392 | 92.79955 |
| BaseFilter% | 93.23074371 | 92.34565127 | 93.16944361 | 92.01997 | 90.60407 | 89.91999 |
| low_quality_reads | 188 | 238 | 1280 | 518 | 756 | 926 |
| too_many_N_reads | 44094 | 43150 | 6304 | 8016 | 8834 | 9730 |
| too_short_reads | 31971550 | 37087894 | 47459850 | 29508066 | 39661496 | 47634182 |
| too_long_reads | 0 | 0 | 0 | 0 | 0 | 0 |
